# Implementation Science to Address Health Disparities During the Coronavirus Pandemic

**DOI:** 10.1089/heq.2020.0044

**Published:** 2020-10-20

**Authors:** Karla I. Galaviz, Jessica Y. Breland, Mechelle Sanders, Khadijah Breathett, Alison Cerezo, Oscar Gil, John M. Hollier, Cassondra Marshall, J. Deanna Wilson, Utibe R. Essien

**Affiliations:** ^1^ Department of Applied Health Science, School of Public Health, Indiana University-Bloomington, Bloomington, Indiana, USA.; ^2^ Department of Applied Health Science, Center for Innovation to Implementation, VA Palo Alto Health Care System, Palo Alto, California, USA.; ^3^ Department of Applied Health Science, Center for Communication and Disparities Research, Department of Family Medicine, University of Rochester, Rochester, New York, USA.; ^4^ Department of Applied Health Science, General Division of Cardiology, Sarver Heart Center General Internal Medicine, University of Arizona, Tucson, Arizona, USA.; ^5^ Department of Applied Health Science, Department of Counseling, Clinical and School Psychology, UC Santa Barbara, Santa Barbara, California, USA.; ^6^ Department of Human Development, College of Community and Public Affairs, Binghamton University, Binghamton, New York, USA.; ^7^ Department of Applied Health Science, Department of Pediatrics, Section of Gastroenterology, Hepatology, and Nutrition, Baylor College of Medicine, Houston, Texas, USA.; ^8^ Department of Applied Health Science, Division of Community Health Sciences, Berkeley School of Public Health, Berkeley, California, USA.; ^9^ Department of Applied Health Science, Division of General Internal Medicine, School of Medicine, University of Pittsburgh, Pittsburgh, Pennsylvania, USA.

**Keywords:** health disparities, knowledge translation, equity, health justice

## Abstract

The coronavirus disease 2019 (COVID-19) pandemic is disproportionally affecting racial and ethnic minorities. In the United States, data show African American, Hispanic, and Native American populations are overrepresented among COVID-19 cases and deaths. As we speed through the discovery and translation of approaches to fight COVID-19, these disparities are likely to increase. Implementation science can help address disparities by guiding the equitable development and deployment of preventive interventions, testing, and, eventually, treatment and vaccines. In this study, we discuss three ways in which implementation science can inform these efforts: (1) quantify and understand disparities; (2) design equitable interventions; and (3) test, refine, and retest interventions.

## Introduction

The novel coronavirus responsible for coronavirus disease 2019 (COVID-19), has infected 38 million individuals worldwide and 7.9 million in the United States as of October 12, 2020 (Ref.^1^). This health crisis has triggered an unprecedented response. From the adoption of preventive strategies, such as physical distancing, to conducting clinical trials testing novel drugs and vaccines, the United States is speeding through the knowledge translation pipeline^2^ faster than ever before. Indeed the development and human testing of vaccines against the novel coronavirus took only 65 days, whereas public health guidelines are revised constantly based on what we learn every day. In this rapid translation, the COVID-19 response is leaving some behind—racial and ethnic minority populations.

National data show African American, Native American, and Hispanic populations are overrepresented among cases of, and deaths from, COVID-19.^1^ Moreover, these disparities are not confined to the treatment and prevention of COVID-19 but expand to other facets of health care, such as access to public health information,^3,4^ telemedicine, and care for chronic conditions.^5^ As the rate of scientific advancement to fight COVID-19 moves quickly, COVID-19 disparities are likely to increase, as we observed during the U.S. H1N1 influenza pandemic.^6^

Implementation science, the scientific study of methods to promote the systematic uptake of evidence-based interventions into routine practice,^7^ can help understand and address the racial and ethnic disparities exposed during the COVID-19 pandemic. Complementing previous perspectives,^8,9^ we discuss three ways implementation science can help build a more equitable COVID-19 response.

## Quantify and Understand Disparities

COVID-19 disparities are exacerbated by the limited data and understanding of their underlying causes. As we write, 47 out of 50 states report COVID-19 infection and death rates by race/ethnicity, whereas only 6 states report COVID-19 testing rates by race/ethnicity.^10^ Furthermore, health centers and insurance plans have limited the publication of these data for the patients under their care. This hinders our understanding of the COVID-19 burden in these communities and our ability to increase the reach of available interventions.

Promising efforts are beginning to emerge. A team from Emory University has built a COVID-19 Health Equity Dashboard, a tool that shows the number of cases and deaths for each U.S. county by age, race, ethnicity, employment status, poverty, and length of commute to the nearest hospital.^11^ The goal of this dashboard is to identify counties with the greatest COVID-19 burden and direct resources toward these communities.

More importantly, efforts should be directed toward unmasking the drivers of these disparities and mitigating the myths of racial biology, behavioral racial stereotypes, and territorial stigmatization.^12^ For this, we need to identify the underlying factors that constrain access to, and benefit from, preventive and curative COVID-19 interventions. For instance, African American, Hispanic, and other minority populations face barriers to accessing and understanding COVID-19 health information, such as language and health literacy limitations.^3,13^ There are also systemic barriers such as the availability of COVID-19 testing, with data from Texas showing testing sites are disproportionately located in whiter communities.^14^ Wide spatial inequities in COVID-19 positivity and incidence in three large metropolitan areas of the United States have also been documented.^15^

Implementation science offers approaches that can help understand factors driving these disparities. For instance, implementation frameworks can be used to understand historical context, values, culture, and needs of minority populations, as demonstrated in a study exploring inequities in hepatitis C care among African American veterans.^16^ Furthermore, behavioral approaches can be used to identify drivers of health behavior and inform the design of interventions; this approach can be used to design interventions for improving sheltering in place, mask wearing, and physical distancing behaviors among minority populations.^17^ Metrics to assess gaps in the reach and adoption of interventions in minority communities and settings are also available.^18^ If we make use of these approaches to improve our understanding of the factors driving health disparities, our chances of reducing inequities will be better.

## Design Equitable Interventions

Efforts to ensure equitable design, implementation, and effectiveness of interventions against COVID-19 are urgently needed. These efforts should include the design of interventions that respond to the culture, history, values, and needs of minority communities. This is crucial since testing, sheltering in place, and physical distancing are not options for many. Furthermore, preventive interventions and testing should be deployed where minority populations live and work, along with strategies to facilitate adoption and implementation in settings that serve these populations. Another critical action is to purposefully sample and include minority populations in studies testing vaccines and treatments to understand their applicability and effectiveness in these populations. Finally, while vaccines and treatments are still in development, now is the time to develop a plan to ensure manufacturing capacity, financing, and timely distribution across minority groups. Indeed, the National Academy of Medicine has issued recommendations to ensure the equitable distribution of the COVID-19 vaccine.

Although not an early focus in implementation science, there are now several frameworks focused on equity,^16,19,20^ which can be used to ensure equitable design, participation, and implementation of interventions. There are also tools to guide the design of interventions based on individual—(e.g., health literacy and cultural beliefs), community—(e.g., clinic physical location), and health system-level characteristics (e.g., access and resource distribution)^21^ to enhance their relevance and potential impact. Approaches to enhance participation and representativeness of minority populations are also available,^18^ as are strategies to enhance adoption, implementation, and sustainability of interventions in settings that serve minority populations.^22^ Implementation strategies can also be used to promote the uptake of clinical guidelines and public health recommendations among health care professionals and decision makers, something crucial as recommendations keep emerging. Overall, implementation science offers approaches to guide the disparity-sensitive design, distribution, and implementation of interventions to fight COVID-19.

Equitable design and implementation are also relevant for the care of chronic conditions that disproportionally affect minority populations. Consider the rapid rollout of telemedicine: early data show that current wide-scale implementation may increase disparities in health care access for vulnerable populations with limited digital literacy or access (e.g., older adults and people with limited English proficiency).^5^ Implementation science can be used to enhance participation and representativeness of minority groups in telemedicine, to explore which telemedicine strategies are preferred (e.g., phone calls and video calls), and to facilitate the adoption of telemedicine in diverse settings. Finally, implementation science can help ensure telemedicine is delivered with fidelity in minority groups, and to promote its sustainability and continued implementation in settings that serve these populations.^18^

## Test, Refine, and Retest

As we deploy COVID-19 preventive and curative interventions, it is critical to assess whether they are working in minority populations. Our current COVID-19 response is failing at this because it models what has been done for other health conditions^23^: interventions have not been designed for, or tested in, racial and ethnic minorities. Also important is the continuous assessment of interventions, as adaptations and refinement may be needed to keep up with the changing nature of COVID-19. Testing and refining of interventions should be guided by comprehensive sociodemographic data to ensure feasibility of implementation, enhance relevance, and improve effectiveness of interventions across racial and ethnic minorities.

Implementation science offers study designs that can be used in this endeavor. For instance, quasi-experimental designs are particularly relevant for testing preventive and treatment approaches in cases when randomization to control conditions is neither be feasible nor ethical.^24^ Pragmatic trials allow for the testing of interventions in real-world settings or routine practice conditions^25^: these can be used to assess whether infection rates change after implementation of testing sites in minority communities, or to examine how infection rates compare with those of communities with no access to testing. Adaptive study designs^26^ allow for testing of interventions that change based on how participants receiving it respond: these can be used to adapt interventions based on the changing needs of minority populations. Step-wedge designs allow the staggered rollout of promising treatments in entire hospitals, settings, or communities that serve minority populations.^2^ The staggered rollout can facilitate implementation of interventions for which resources are scarce, and can help stop rollout of interventions that are not working in minority populations, avoiding waste of resources.

**FIG. 1.**
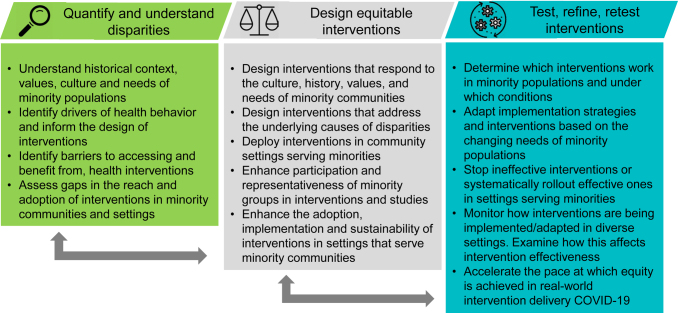
Examples of how implementation science can be used to address COVID-19 disparities. This figure highlights three ways in which implementation science can help address COVID-19 disparities: (1) quantify and understand the gap; (2) design equitable interventions; and (3) test, refine, and retest interventions to optimize for minority populations. The bidirecional arrows indicate information obtained in each step influences activities and decisions in previous or subsequent steps. This also depicts an iterative process in which understanding and addressing disparities may require going back and forth between these steps. COVID-19, coronavirus disease 2019.

Finally, hybrid effectiveness–implementation study designs can be used to identify and address underlying factors driving disparities in both effectiveness and implementation of interventions.^27^ Specifically, there are three types of hybrid study designs that allow the simultaneous testing/assessment of intervention effectiveness and the testing/assessment of intervention implementation. These hybrid study designs can be used to accelerate the implementation of effective interventions in settings serving minority communities. The examples outlined here are summarized in Figure 1.

## Conclusion

The national COVID-19 response should address, not exacerbate, health disparities. As we speed through the knowledge translation pipeline, we have a unique opportunity to use implementation science and address the racial and ethnic health disparities COVID-19 has exposed. Successfully addressing disparities amid a global pandemic will ensure that not only the most vulnerable but also all individuals have access to, and benefit from, quality health care and public health interventions. Although we focus on COVID-19, the approach we outline here could also help tackle health disparities across several conditions. The lessons are clear and opportunities to tackle ingrained health injustices in this country are paramount.

## Abbreviation Used

COVID-19: coronavirus disease 2019
